# A structural model of the *E. coli *PhoB Dimer in the transcription initiation complex

**DOI:** 10.1186/1472-6807-12-3

**Published:** 2012-03-20

**Authors:** Chang-Shung Tung, Benjamin H McMahon

**Affiliations:** 1 Theoretical Biology & Biophysics, Los Alamos National Laboratory, Los Alamos, NM 87545, USA

## Abstract

**Background:**

There exist > 78,000 proteins and/or nucleic acids structures that were determined experimentally. Only a small portion of these structures corresponds to those of protein complexes. While homology modeling is able to exploit knowledge-based potentials of side-chain rotomers and backbone motifs to infer structures for new proteins, no such general method exists to extend our understanding of protein interaction motifs to novel protein complexes.

**Results:**

We use a Motif Binding Geometries (MBG) approach, to infer the structure of a protein complex from the database of complexes of homologous proteins taken from other contexts (such as the helix-turn-helix motif binding double stranded DNA), and demonstrate its utility on one of the more important regulatory complexes in biology, that of the RNA polymerase initiating transcription under conditions of phosphate starvation. The modeled PhoB/RNAP/σ-factor/DNA complex is stereo-chemically reasonable, has sufficient interfacial Solvent Excluded Surface Areas (SESAs) to provide adequate binding strength, is physically meaningful for transcription regulation, and is consistent with a variety of known experimental constraints.

**Conclusions:**

Based on a straightforward and easy to comprehend concept, "proteins and protein domains that fold similarly could interact similarly", a structural model of the PhoB dimer in the transcription initiation complex has been developed. This approach could be extended to enable structural modeling and prediction of other bio-molecular complexes. Just as models of individual proteins provide insight into molecular recognition, catalytic mechanism, and substrate specificity, models of protein complexes will provide understanding into the combinatorial rules of cellular regulation and signaling.

## Background

Solving structures of complexes is inherently more difficult than solving those for individual proteins. As a result, significantly fewer structures of protein complexes than individual proteins have been determined experimentally [1]. In recent years, homology modeling [2,3] proved to be successful when the target protein has a similar sequence to proteins with known structures. However, the lack of a sufficiently large database of reference complexes makes the method unsuitable for structural modeling of protein complexes. A conceptually simple and straightforwardly applicable approach for modeling structures of bio-molecular complexes is highly desirable. When proposing new protein complexes, the models developed should be checked against the following attributes: stereo-chemically sound, having sufficient interfacial Solvent Excluded Surface Areas [4] (SESAs) to provide adequate binding strengths, physically meaningful for transcription regulation and consistency with the known experimental data.

PhoB is a response regulator of the two-component signaling system that is activated under phosphate starvation conditions [5]. It activates more than 30 genes of the *pho *regulon [6]. Structurally similar to many other response regulators, PhoB has two domains: an N-terminal Receiver Domain (RD) and a C-terminal Effector Domain (ED). The ED of PhoB adopts a winged-helix structure that consists of three α-helices flanked by two sets of β-sheets [7]. The PhoB RD adopts a β-α structure [8] that can be classified as a flavodoxin-like fold according to SCOP [9]. The flavodoxin-like fold can be found in RDs of other response regulators as well as flavodoxins [10], cytochrome-P450 oxidoreductase [11] and Toll/Interleukin Receptor TIR domains [12]. These protein domains share the same structural fold with little or no sequence homology.

While PhoB has long been known to regulate the expression of the *pho *regulon, the specific geometry of the transcription initiation complex remains undetermined. In recent years, a significant amount of work has been dedicated to solving structures of RNAP complexes (see review articles [13-15]). The bacterial RNA polymerase (RNAP) is a multi-molecular complex consisting of five subunits including: two α-subunits, a β-subunit, a β'-subunit and an ω-subunit. To start transcription, the RNAP has to first bind a σ-subunit. This RNAP/σ-subunit complex then recognizes and binds to a targeted DNA operator site to go through the transcription process. In 2002, the low-resolution (6.5 Å) structure of the *Thermus aquaticus *RNAP holoenzyme with a fork-junction promoter DNA complex (PDB accession code: 1L9Z) was solved [16]. Since then, crystal structures of different RNAP holoenzymes were solved to a higher resolution [17,18] (e.g., PDB accession codes: 1ZYR, 2A6E). More recently, an electron microscopy (EM)-derived structure of a Catabolite Gene Activator (CAP)-dependent transcription initiation complex has been derived [19] (PDB accession code 3IYD). The structural information available so far provides a knowledge base for modeling of the transcription initiation complex together with the response regulator PhoB. In particular, the structure of the Catabolite Gene Activator (CAP)-dependent transcription initiation complex (3IYD) provides an ideal template for modeling structure of the PhoB-dependent transcription initiation complex.

## Results and discussion

**Figure 1:**
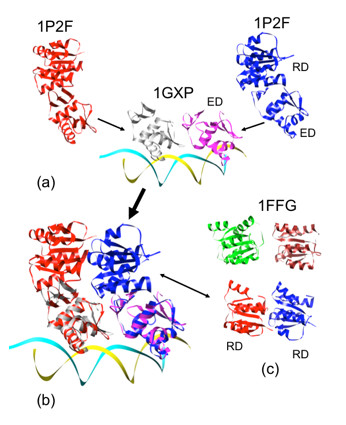
**Structural model of the PhoB dimeric complex binding to its targeted DNA duplex**. Matching ED of DrrB to ED of PhoB is shown in 1a. The resultant PhoB/DNA model is shown in 1b. RD/RD Motif Binding Geometry (MGB) in CheY (PDB accession code: 1FFG) is similar to that in the modeled PhoB, and is shown in 1c.

We begin by considering the PhoB dimer as it interacts with DNA, for which no complete structure exists. In the crystal structure of the PhoB ED dimer bound to *pho *box DNA (PDB accession code: 1GXP[7], shown as magenta and white molecules in Figure 1a), the binding of DNA direct repeats force the ED dimer to bind with a tandem symmetry. The known structure of the PhoB RD dimeric complex [8] (PDB accession code: 2JB9), however, follows a two-fold rotational symmetry. While it is possible to simply rotate one of the EDs relative to the RD to make a complex satisfying both structures, this procedure results in a tightly stretched linker, asymmetry between the two PhoBs, and fabricating an RD-ED interface from scratch. Alternatively, we examine the variety of response regulator structures that contain RD and ED together (PDB accession codes: 1KGS, 1P2F, 1YS6, 2GWR, 2OQR, 1A04, 1YIO). These structures contain the information of RD/ED MBGs and demonstrate that the two domains can interact with a variety of binding geometries.

Combining the information of RD/ED MBGs with the structure of the ED/ED dimeric complex (1GXP), we explore the potential solutions for the PhoB dimeric complex. Out of the RD/ED conformations, only that of DrrB [20] (1P2F, shown as the red and the blue molecules in Figure 1), a PhoB/OmpR homolog, provides a satisfactory solution where the two RDs are in contact but not overlapping. Combining the structural information of ED/ED (1GXP), RD/ED (1P2F), ED (1GXP) and RD (2JB9), the model of the PhoB dimeric complex is developed (shown as the white and magenta molecules bound to DNA in Figure 1b). This model structure has appealing features including: good stereochemistry (no clashes between domains, stable interface surface area), protein-like structure (contents of secondary structures, density, etc.) and several of the known MBGs.

This PhoB in the modeled complex contains a previously unseen interface between RDs, however, because of the tandem head-to-tail orientation - that is different from the two-fold symmetry observed in the PhoB RD/RD dimer (2JB9). The next question is "does the new MBG between the two RDs in the model exists in other protein domains of a similar fold?" To answer this question, we search for interfaces between domains that have the flavodoxin-like fold and give the two domains with a tandem symmetry. Interestingly, the CheY (a chemotaxis protein) of the two CheY-P2 heterodimers in the crystal asymmetric unit [21] (PDB accession code: 1FFG), has the two flavodoxin-like molecules following a tandem symmetry. This contact of the two CheYs (1FFG) in the crystal is very similar to that of the PhoB dimeric RDs as shown in Figure 1c. While this particular CheY dimeric arrangement may not be functionally relevant for the CheY-CheA interaction, it does provide a potential MBG for the interaction of flavodoxin-like molecules.

**Figure 2:**
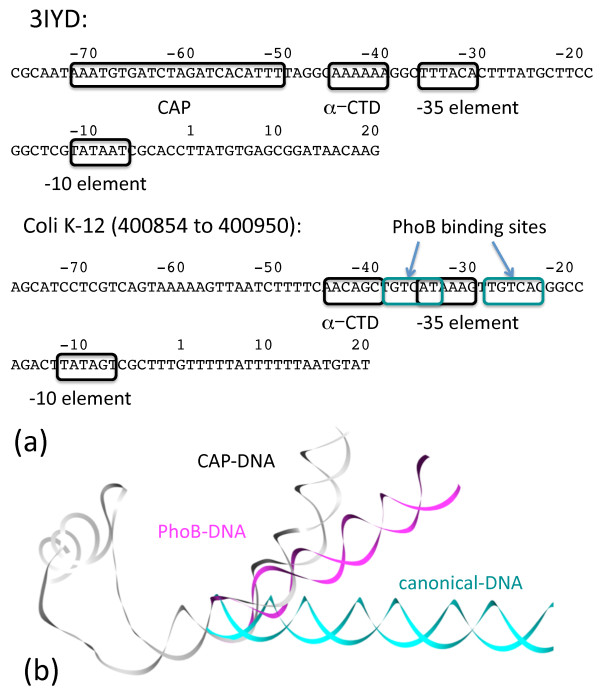
**The sequences of the *E. coli *CAP-dependent and *PhoB *promoters with the corresponding protein binding sites indicated are shown in 2a**. 2b shows that CAP and PhoB bind and bend the DNA to a different degree than the canonical DNA.

We turn our attention to the transcription initiation complex. We choose to use the transcription initiation complex with DNA and the Catabolite Activator Protein bound to it (PDB ID: 3IYD) as a template for our model. The DNA duplex can serve as a structural link and allow the assembly of all the components into one functional unit. All the proteins in the complex either have a direct contact (i.e., α-subunit, σ-subunit, PhoB) or contacts thru other molecules (i.e., β-subunit, β'-subunit, ω-subunit) that can link to the DNA molecule. The DNA molecule that we select for this study is the *E. coli *K-12 PhoA promoter (400854 to 400950 bp) with both σ-subunit and PhoB binding sites (information derived from RegulonDB [22]). To enable comparison, the sequences of the two promoters (CAP and PhoA) are shown in Figure 2a with the CAP promoter (as found in 3IYD) shown on the top and the PhoA promoter shown at the bottom. The protein binding sites on the two promoters are highlighted in boxes. The main difference between the two promoters is the relative binding locations for the two factors. The CAP binding sites are located upstream of the -35 site while the PhoB binding sites are overlapping with the -35 sites. There was a structural concern, whether the -35 and the two PhoB binding sites can be utilized simultaneously. When these binding sites are utilized simultaneously, a set of interactions between the RNAP and the two PhoB molecules can be predicted by our model.

In additional to the difference in the binding sites, changes in the DNA from 3IYD will be required because the CAP dimer binds and bends the DNA promoter much more than does the PhoB dimer. Therefore, the promoter region of the DNA in the PhoB transcription initiation complex has to be remodeled from the template structure (3IYD). Using a "motif modeling approach" as described in our earlier work [23], the structure of the DNA upstream to this overlapping region (including the PhoB binding sites) can be modeled using the structure of DNA from the PhoB ED/DNA complex (from 1GXP). This promoter DNA is extended upstream with a piece of canonical DNA duplex to accommodate the α-subunit C-terminal domain (CTD) binding. As a comparison, we have modeled the same piece of DNA upstream to this overlapping region using only a piece of canonical DNA B-duplex. The template DNA (from 3IYD), the remodeled promoter DNA for PhoB transcription initiation complex, and the upstream DNA in a canonical B-duplex conformation are shown in Figure 2b in white, magenta, and cyan respectively.

**Figure 3:**
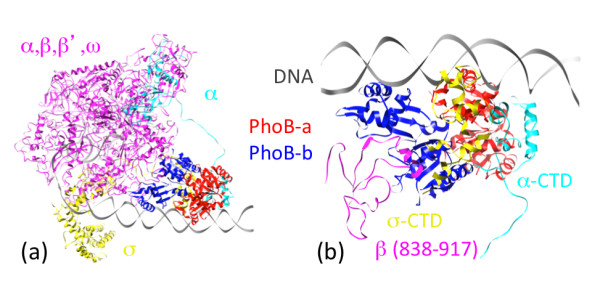
**The modeled structure of the PhoB dimer in the transcription initiation complex**. The color-coding of different components in the complex is shown in 3a. The α,β,β',ω subunits are drawn in magenta, the α-subunit that interacts with PhoB is drawn in cyan, the σ-subunit is drawn in yellow, the PhoB-a is drawn is red and the PhoB-b is drawn in blue. Figure 3b shows the close-up of molecules (α-CTD, σ-CTD and segment of β-subunit) interacting with the two PhoB molecules.

After the structure of the promoter DNA duplex is re-modeled, the corresponding proteins can be assembled back into the PhoB transcription initiation complex using the information of their MBGs with their targeted sites on the DNA (Additional file 1). With the remodeling of the promoter DNA, the positions and orientations of α-CTD and σ-CTD are different from those in the template structure. The connecting loops between the N-terminal domain (NTD) and CTD of the α- and σ-subunits also needed to be changed accordingly [24]. The resultant structure (shown in Figure 3a) has the subunits interacting but not overlapping with each other, a necessary condition for complex structural modeling. According to the model, α-CTD, σ-CTD as well as a segment (residues 839 to 917) of β-subunit are in direct contact with the two PhoB molecules in the complex. To improve the stereochemistry between the interacting subunits, the remodeled portions of the complex, including the DNA promoter, the PhoB dimer, the α-CTD, the σ-CTD and residues 839 to 917 of β-subunit were subjected to a refinement procedure using AMBER [25].

**Figure 4:**
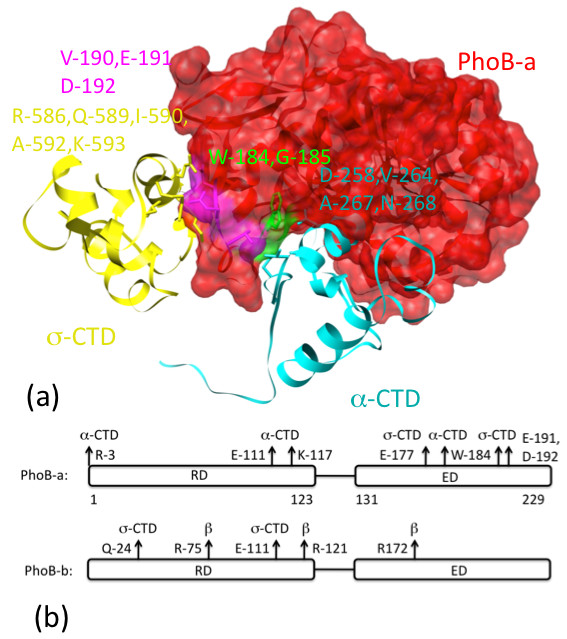
**Interactions between PhoB molecules and subunits (α-, β- and σ-subunits) of the polymerase complex**. In Figure 4a, PhoB-a is drawn in both ribbon and transparent surface plots while the α- and σ-CTD are drawn in ribbon plots. The residues involved in binding are highlighted. In Figure 4b, the residues on the two PhoB molecules interacting with the subunits of the polymerase are highlighted with arrows.

The energy-refined structure of this portion of PhoB transcription initiation complex is shown in Figure 3b and a coordinate file is available as supplementary material. The clearest self-consistency check from our model is that the overlapping binding sites covering the -35 region allow the simultaneous binding of the PhoB dimer and the σ-CTD without violating the volume exclusions for all the molecules involved in the binding. Both α-CTD and σ-CTD interact directly with one of the PhoB molecules (shown in red in Figure 3) that binds to the site upstream of the -35 region. For a more detailed check on the validity of our model, we note that the residues at the interface between these molecules include: R-586, Q-589, I-590, A-592, K-593 from the α-CTD, D-258, V-264, A-267, N-268 from the σ-CTD and W-184, G-185, V-190, E-191, D-192 from the PhoB (as highlighted in Figure 4). This result is consistent with the four PhoB residues (W-184, G-185, V-190 and D-192) identified to be involved in the polymerase binding based on mutation study [26]. The residues on the two PhoB molecules that interact directly with α-CTD, β-subunit and σ-CTD are annotated in Figure 4b. Our results indicate that both the RD and ED domains of the two PhoB molecules in the dimer are interacting with the RNAP/σ-subunit of the transcription initiation complex. The Solvent Excluded Surface Areas for PhoB-a/α-subunit, PhoB-a/σ-subunit and PhoB-b/β-subunit are 2,867 Å^2^, 1,098 Å^2 ^and 2,165 Å^2 ^respectively. These values are consistent with those (639 Å^2 ^to 3,228 Å^2^) [27] observed in the heterocomplexes from PDB.

There exist off-the-shelf software that allows dockings of proteins or protein domains into complexes/full proteins (e.g., ZDOCK [28], AutoDock [29], RosettaDock [30]). These programs apply different sampling approaches and scoring functions with various degrees of success (e.g., see CAPRI [31] assessments). These docking procedures seem to work at their best if the interaction between the components is strong and/or there exists a global binding minimum. As a quick comparison, we have downloaded one of these programs, ZDOCK, and generated 2,000 structures (MBGs) docking the two domains RD (2JB9, residues 3-123) and ED (1GXP, residues 127-229) for deriving the PhoB structure. The two domains (RD & ED) of PhoB molecule are separated by a loop of 4-peptides group. There is a physical limitation for a 4-residues loop to make the connection. If the cut-off length for a 4-residues loop is set to be 14 Angstrom (approximately corresponds to a complete extended conformation), only 2.12% (43) of the 2,000 MBGs satisfied the connection criteria. If we focus on the set of the top 100 MBGs, structures 21 and 96 are the two that allow the RD-ED connection. A further look at the PhoB-PhoB dimer structures modeled based on the two ED-RD MBGs and the structure of the ED-ED-DNA complex (1GXP), neither structure is stereochemically feasible due to the domain overlapping including clashes between protein-protein and protein-DNA. If all the MBGs of the two domains from the docking study are compared to the MBG from our model, the closest came from structure 1,934 with a RMSD of 4.0 Angstrom (based on C_α _atoms only). Overall, the docking procedure is less than efficient (only ~2% of the docked structures satisfies the connectivity constraint). It was also found that the selection of the relevant PhoB structure out of the pool of a large number of potential MBGs from the docking study is a non-trivial task.

## Conclusion

We have demonstrated that Motif Binding Geometry (MBG) can be used to model structure of the PhoB dimer as it interacts with the transcription initiation complex (PhoB/RNAP/DNA) *of E. coli*. While the limited space available for the targeted protein in the molecular complex makes the modeling of the protein structure more challenging, it also provides a stringent test for choosing the relevant structure from the pool of potential conformations. While the two domains (ED and RD) of PhoB adopt a different symmetry when crystallized, it is not obvious how to assemble the PhoB dimer from the information of its domain structures. Using the excluded volume information and known MBGs between the ED and RD, we are able to develop a structural model for the PhoB dimeric complex where the two RD domains follow a tandem symmetry similar to that as seen in the two flavodoxin-like folds of CheY, a chemotaxis protein. The modeled PhoB dimer can bind to the direct repeat *Pho *box in the promoter region and interact directly with the α-, β- and σ- subunits of the RNAP.

Just as protein structures serve to integrate a variety of biochemical information and advance our understanding of the enzymatic reactions and molecular machines that enable life to continue, modeling of protein complexes will shed light on the protein interaction networks responsible for regulatory and signaling processes of cells. While our approach has not yet been tested with other protein complexes, it is hoped that the reader will see our methodology as a way of integrating the evolutionary, physical, and biological experimental data to produce new, testable, hypothesis.

## Methods

### Motif Binding Geometry (MBG) used for complex homology modeling

Upon binding, the folds of proteins often remain unchanged while the specifics of the surface may be adjusted to accommodate the interactions. Therefore, while docking of molecules by matching surface shape is an attractive method in principle, significant errors can be introduced into the overall binding geometry if induced fitting at the interface is involved during the binding process. Here, we introduce a structural based concept for bio-molecular docking by matching the scaffoldings (secondary structural motifs) of the interacting molecules to those with homologous folds and known MBGs. This approach is useful to structural modeling both to arrange stable folded domains in the intact protein and to find geometries of individual molecules in the complex. The method can readily provide a manageable set of potential solutions for further study and/or refinement.

### Motif structural matching

Protein motifs consists of secondary structural elements (α-helix and β-sheet) arranged with a specific geometry in space. In cases where sequence homology is low (e.g., < 20% identity), it is difficult to discern structural alignments using only sequence alignments. A general approach based on the structural information is required for motif structural matching. We use the secondary structural elements to align the motifs. When each of the secondary structural elements is represented by a line vector, the structural matching can be accomplished by minimizing the angles (θ) and the minimum distances (*d*) between the set of corresponding line vectors. The Metropolis Monte Carlo simulation [32] is used for the minimization procedure.

### Graphics

Molecular graphics images were produced using the UCSF Chimera package [33] from the Resource for Biocomputing, Visualization and Informatics at the University of California, San Francisco.

## Competing interests

The authors declare that they have no competing interests.

## Authors' contributions

CST designed and carried out the modeling works for the PhoB dimer and the PhoB dimer in the transcription initiation complex. BHM has regularly participated in discussions throughout the course of the study. Both CST and BHM are involved in the preparation of the manuscript. All authors read and approved the final manuscript.

## Supplementary Material

Additional file 1 PDB format coordinate file of the modeled complex. Click here for file

## References

[B1] Berman HM The protein data bank Nucleic Acids Res 2000 28 235 242 10.1093/nar/28.1.235 10592235 PMC102472

[B2] Marti-Renom MA Comparative protein structure modeling of genes and genomes Annu Rev Biophys Biomol Struct 2000 29 291 325 10.1146/annurev.biophys.29.1.291 10940251

[B3] Ginalski K Comparative modeling for protein structure prediction Curr Opin Struct Biol 2006 16 172 177 10.1016/j.sbi.2006.02.003 16510277

[B4] Hubbard SJ Thornton JM NACCESS, Version 2.1, Department of Biochemistry and Molecular Biology University College, London

[B5] Makino K Nucleotide sequence of the PhoB gene, the positive regulatory resolution J Mol Biol 1986 1986 190 37 44 3537313 10.1016/0022-2836(86)90073-2

[B6] Kim SK Dual transcriptional regulation of the *Escherichia col *phosphate-starvation-inducible *psi *gene of the phosphate regulon by PhoB and the cyclic AMP (cAMP)-cAMP receptor protein complex J Bacteriol 2000 182 5596 5599 10.1128/JB.182.19.5596-5599.2000 10986267 PMC111007

[B7] Blanco AG Tandem DNA recognition by PhoB, a two-component signal transduction transcriptional activator Structure 2002 10 701 703 10.1016/S0969-2126(02)00761-X 12015152

[B8] Arribas-Bosacoma R The x-ray crystal structures of two constitutively active mutants of the Escherichia coli PhoB receiver domain give insights into activation J Mol Biol 2007 366 626 641 10.1016/j.jmb.2006.11.038 17182055 PMC1855202

[B9] Murzin AG SCOP: a structural classification of proteins database for the investigation of sequences and structures J Mol Biol 1995 247 536 540 7723011 10.1006/jmbi.1995.0159

[B10] Fukuyama K Matsubara H Crystal structure of oxidized flavodoxin from a red alga *Chondrus crispu *refined at 1.8-Å resolution J Mol Biol 1992 225 775 789 10.1016/0022-2836(92)90400-E 1602481

[B11] Hubbard PA NADPH-cytochrome P450 oxidoreductase J Biol Chem 2001 276 29163 29170 10.1074/jbc.M101731200 11371558

[B12] Tao X An extensively associated dimmer in the structure of the C713S mutant of the TIR domain of human TLR2 Biochem Biophys Res Comm 2002 299 216 221 10.1016/S0006-291X(02)02581-0 12437972

[B13] Bourkhov S Nudler E RNA polymerase holoenzyme: structure, function and biological implications Curr Opin Microbiol 2003 6 93 100 10.1016/S1369-5274(03)00036-5 12732296

[B14] Browning DF Busby SJW The regulation of bacterial transcription initiation Nat Rev Microbiol 2004 2 57 65 10.1038/nrmicro787 15035009

[B15] Vassylyev DG Artsmovitch I Tracking RNA polymerase one step at a time Cell 2005 123 977 979 10.1016/j.cell.2005.11.030 16360025

[B16] Murakami KS Structural basis of transcription initiation: an RNA polymerase holoenzyme-DNA complex Science 2002 296 1285 1290 10.1126/science.1069595 12016307

[B17] Tuske S Inhibition of bacterial RNA polymerase by streptolydigin: Stabilization of a straight-bridge-helix active-center conformation Cell 2005 122 541 552 10.1016/j.cell.2005.07.017 16122422 PMC2754413

[B18] Artsimovitch I Allosteric modulation of the RNA polymerase catalytic reaction in an essential component of transcription control by rifamycins Cell 2005 122 351 363 10.1016/j.cell.2005.07.014 16096056

[B19] Hudson BP Three-dimensional EM structure of an intact activator-dependent transcription initiation complex Proc Natl Acad Sci USA 2009 106 19830 19835 19903881 10.1073/pnas.0908782106 PMC2775702

[B20] Robinson VL Wu T Stock AM Structural analysis of the domain interface in DrrB, a response regulator of the OmpR/PhoB subfamily J Bacteriol 2003 185 4186 4194 10.1128/JB.185.14.4186-4194.2003 12837793 PMC164896

[B21] Gouet P Further insights into the mechanism of function of the response regulator CheY from crystallographic studies of the CheY-CheA_124-257 _complex Acta Crystallogr sect D 2001 57 44 45 10.1107/S090744490001492X 11134926

[B22] Huerta AM RegulonDB: A database on transcription regulation in Escherichia coli Nucleic Acids Res 1998 26 55 60 10.1093/nar/26.1.55 9399800 PMC147189

[B23] Tung CS All-atom homology model of the *Escherichia col *30S ribosomal subunit Nat Struct Mol Biol 2002 9 750 755 10.1038/nsb841 12244297

[B24] Byu K Modulation of high affinity hormone binding J Biol Chem 1998 273 6285 6291 10.1074/jbc.273.11.6285 9497355

[B25] Case DA The Amber biomolecular simulation programs J Comput Chem 2005 26 1668 1688 10.1002/jcc.20290 16200636 PMC1989667

[B26] Makino K DNA binding of PhoB and its interaction with RNA polymerase J Mol Biol 1996 259 15 26 10.1006/jmbi.1996.0298 8648643

[B27] Jones S Thornton JM Principles of protein-protein interactions Proc Natl Acad Sci USA 1996 93 13 20 10.1073/pnas.93.1.13 8552589 PMC40170

[B28] Pierce B M-ZDOCK: A Grid-based approach for Cn Symmetric Multimer Docking Bioinformatics 2005 21 1472 1476 10.1093/bioinformatics/bti229 15613396

[B29] Trott O AutoDock Vina: improving the speed and accuracy of docking with a new scoring function, efficient optimization and multithreading J Comput Chem 2010 31 455 461 19499576 10.1002/jcc.21334 PMC3041641

[B30] Gray JJ Protein-Protein docking with simultaneous optimization of rigid-body displacement and side-chain conformations J Mol Biol 2003 331 281 299 10.1016/S0022-2836(03)00670-3 12875852

[B31] Janin J CAPRI: a critical assessment of predicted interactions Proteins 2003 52 2 9 10.1002/prot.10381 12784359

[B32] Metropolis N Equation of state calculations by fast computing machines J Chem Phys 1953 21 1087 1092 10.1063/1.1699114

[B33] Pattersen EF UCSF Chimera--a visualization system for exploratory research and analysis J Comput Chem 2004 25 1605 1612 10.1002/jcc.20084 15264254

