# Supplementary material for: A structural model of the E. coli PhoB Dimer in the transcription initiation complex
Source: BMC Struct Biol. 2012 Mar 20;12:3. doi: 10.1186/1472-6807-12-3 (PMC3348028; doi:10.1186/1472-6807-12-3)
Supplement: Additional file 1 — PDB format coordinate file of the modeled complex. [file 1472-6807-12-3-S1.ZIP › index.html]

The structural model of the E. coli PhoB dimer in the transcription
initiation complex.
  
There are two coordinate files:

The first file, RNAP.model.pdb, contains the model of the following molecules:

- the PhoB dimer (strands P & Q),
  
 - one alpha subunit of the polymerase (strand A),
  
 - the sigma subunit of the polymerase (strand F),
  
 - the two complimentary strands of the DNA with the promoter and PhoB
binding sites (strand I & J).

The second file,  RNAP.3iyd.pdb, has the remaining molecules in the complex.
The structures of these molecules are obtained from the EM derived
model of the E. coli transcription initiation complex (PDB accession
code: 3IYD). These molecules are:

- one alpha subunit of the polymerase (strand B),
  
 - the beta subunit of the polymerase (strand C),
  
 - the beta prime subunit of the polymerase (strand D),
  
 - the omega subunit of the polymerase (strand E).
